# Comparing the Knowledge and Awareness of Dental Students, Dental Practitioners and Patients About Botulinum Toxin and Its Therapeutic Applications in Temporomandibular Disorders

**DOI:** 10.1155/ijod/6887125

**Published:** 2025-10-15

**Authors:** Joshua Gower, Nihal Nayak, Jack Troy, Kobi Rutherford, Sanjay Vasudeva, Mahmoud M. Bakr

**Affiliations:** General Dental Practice, School of Medicine and Dentistry, Griffith University, Gold Coast, Queensland, Australia

**Keywords:** botulinum toxin, dental practitioners, dental students, patients, questionnaire, temporomandibular disorders, therapeutic applications

## Abstract

**Background:**

This study aims to assess the awareness of botulinum toxin (BTX), a treatment for temporomandibular disorders (TMDs) and bruxism, among patients, dental students and dental practitioners.

**Methods:**

Over a 7-month period (September 2023–March 2024), a paper-based questionnaire was distributed to randomly selected patients, dental students, and practitioners at the Griffith University Dental Clinic (GUDC) in Queensland, Australia. The questionnaire assessed participants' knowledge and awareness of BTX as a treatment for TMD and bruxism.

**Results:**

A total of 325 participants completed the questionnaire, comprising 171 patients, 130 students and 24 dental practitioners. Dental practitioners demonstrated the highest knowledge and awareness surrounding the uses of BTX in TMD treatment. While students had less knowledge than practitioners, their understanding of BTX increased as they advanced through their studies. Both dental practitioners and students favored non-BTX treatments for TMD. Patients exhibited limited awareness of BTX, with female patients being more informed and receptive.

**Conclusion:**

The results from this study highlight the need for increased awareness of BTX for the treatment of TMD, particularly amongst patients and dental students, and to a lesser extent, dental practitioners.

## 1. Introduction

Botulinum toxin (BTX) is a biological neurotoxin produced by the bacterium *Clostridium botulinum* which exerts its effects through a highly specific mechanism targeting the neuromuscular junction [1]. This innate ability of BTX to induce muscular paresis has promoted its multifunctional use within the modern cosmetic and medical fields [2]. These uses include the treatment of chronic migraines, cervical dystonia, spasticity, adult bladder dysfunction, masseteric hypertrophy, rhytids and other various cosmetic and therapeutic treatments [3–9].

One of these therapeutic treatments also involves the treatment of temporomandibular disorders (TMDs) with BTX. The temporomandibular joint (TMJ) can experience extrinsic or intrinsic issues that may lead to pain; which are known as TMD [10, 11]. Studies suggest that approximately 60%–70% of people experience one or more signs of TMD [12], however, the prevalence of TMD within the Australian population is estimated to be 23% [13]. Despite these statistics, TMD is still poorly understood, and the origin is hypothesised to be multifactorial. A review by List and Jensen [14] concluded that the aetiology of TMDs is complex, relating to multiple risk factors. This may illustrate that the masticatory muscles play a large role in TMD. Therefore, the ability of BTX to paralyse or weaken muscular activity may be beneficial for bruxism and the reduction of TMD symptoms [2, 15].

A concomitant disorder frequently experienced by individuals with TMD and facial pain is bruxism. Bruxism is a parafunctional habit of grinding or clenching that can occur when awake or asleep [16]. Recent studies have illustrated that the therapeutic use of BTX can reduce orofacial pain, bite force, function, increase the maximum mouth opening and decrease muscular strength including the muscles of mastication in TMD and bruxism patients [17, 18]. A systematic review by Ramos-Herrada et al. [19], and a meta-analysis by Li et al. [20] found that BTX serotype A injections were a safe and effective treatment for the relief of muscular-related orofacial pain and joint dysfunction. BTX was found to be effective at reducing orofacial pain; regardless of the dosages used and was equally effective as oral appliances [20]. Another study by Hosgor and Altindis [21] stated that BTX injections into the masseter and temporalis muscles of patients suffering from sleep bruxism decreased pain levels by 59% and 70% after 1 month and 6 months, respectively.

A review of current literature suggests that BTX is an effective treatment for improving function and alleviating pain related to TMD and bruxism. However, there is a notable lack of literature regarding the education and confidence of practitioners and students using BTX for TMD treatment in Australia. Similarly, there is limited research into the awareness of BTX for TMD and bruxism within a patient population. Even more sparse in the literature is the impact of certain demographic variables on BTX awareness. This accentuates the need for further research on the awareness of the uses of BTX as a treatment option within the dental community.

This study aims to evaluate the current knowledge and awareness regarding the use of BTX to treat TMD and bruxism amongst dental students, dental practitioners and patients within the Griffith University Dental Clinic (GUDC), while also comparing the levels of awareness between these groups. Therefore, the null hypothesis is that there is no significant difference in the awareness levels of BTX as a treatment for TMD among and between patients, dental students, and dental practitioners at GUDC. The alternate hypothesis is that a significant difference will be observed between participant groups.

## 2. Materials and Methods

### 2.1. Study Design

This study utilises a cross-sectional study design to assess the awareness and attitude of GUDC patients, students and dental practitioners towards the use of BTX in the treatment of TMD and bruxism. The study was approved by the Griffith University Human Research Ethics Committee (GU Ref No: 2023/780) on the 26th of September 2023. Data collection were conducted over 7 months and commenced in September 2023 and was completed in March 2024. The sample size represented the population, which was calculated to be 252 participants according to the G^*⁣*^*∗*^^Power software, with a confidence interval of 95%. It was determined that 150 patients, 100 students and 20 dental practitioners would be required from GUDC to complete the questionnaires.

Individuals were eligible to participate in this study if they were either a third-, fourth- or fifth-year dental student enrolled in the Bachelor of Dental Health Science/Masters of Dentistry at Griffith University; or they were a registered dentist/dental specialist; or a member of the public attending the GUDC.

### 2.2. Questionnaires

Three separate questionnaires were developed for each respective group to assess the awareness and attitudes towards the use of BTX for the treatment of TMD and bruxism. The patient questionnaire consisted of three demographic questions, including their age, gender and education level. Further, six dichotomous questions (yes/no) related to their experience, willingness and education surrounding BTX. There was also the inclusion of two written response questions that assessed previous experience of TMD and their willingness to receive BTX treatment. The dental student questionnaire consisted of three demographic written response questions, including age, gender and year level. With another, six dichotomous (yes/no) questions assessing general knowledge of BTX and attitudes towards furthering education on BTX, and two written response questions assessing the correct dosage of BTX and preferred treatment for TMD and bruxism.

The dental practitioner questionnaires consisted of four demographic written response questions, including age, gender, years of experience and specialty type. Additionally, seven dichotomous (yes/no) questions assessing BTX knowledge, BTX training received, clinical experience administering BTX, and one 5-point Likert scale (with 1 representing strongly disagree and 5 representing strongly agree), which self-assessed dental practitioners' confidence in delivering BTX as a treatment, were included. Three dichotomous (yes/no) questions relating to awareness of BTX for non-cosmetic use, BTX side effect awareness, and duration of BTX effect overlapped in each questionnaire to allow comparison between groups in the analysis.  Table 1 summarises the questions that were used in each questionnaire. It should be noted that the dental practitioners at the university clinic were either academics from the public sector that undergo clinical practice privately within the private university suites or dental practitioners from the private sector that are engaged on a sessional/casual basis to teach within the university for one or 2 days per week. Therefore, they were representative of the different sectors of the dental industry.

Following completion, the questionnaires were given to three patients, three students and three dental practitioners at GUDC to assess the clarity and quality of the questions. Following feedback, the survey was modified to improve clarity and was deemed satisfactory by these participants. The average time for survey completion was approximately 5 min. The data obtained from the pilot testing of the questionnaires did not show any abnormal patterns or outliers which confirmed the validity, reliability and clarity of the questionnaires. Data collection then commenced, and the survey was completed by participants under supervision from the research team in the GUDC or GUDC waiting room, and participants were made aware that participation was voluntary. The inclusion criteria for students were current enrolment in 3rd year of Bachelor of Dental Health Science or 1st or 2nd year of Masters of Dentistry (4th or 5th-year dental students, respectively), for dental practitioners it was current employment by GU, and for patients, it was any current patient attending GUDC. Subjects were approached at random to participate. The participation rates were approximately 90% for students and patients, and approximately 80% for dental practitioners, all of whom participated voluntarily after providing informed consent.

### 2.3. Data Analysis

Group-specific dichotomous-based responses were subjected to chi-square analysis, along with the three dichotomous questions that overlapped between the patients, students and dental practitioners. Data analysis were completed using IBM SPSS Statistics Version 29. Chi-square analysis was deemed an appropriate statistical analysis test due to the categorical nature of the variables.

The discrete demographic variable, age, was collected for all participants and then categorised into age groups for analysis (Table 2). At a student and dental practitioner level, written responses regarding ‘what treatment would you prefer for TMD and/or bruxism?' were recorded as qualitative data which was transformed to a quantitative code through data categorisation (Table 3) and then subject to analysis via chi-square tests.

The confidence of dental practitioners in delivering BTX was evaluated through the question, ‘Would you feel comfortable delivering botulinum toxin?' using a Likert scale with response options being categorised from one to five (‘strongly disagree,' ‘disagree,' ‘neutral,' ‘agree,' ‘strongly agree,' respectively). This data was then subjected to chi-squared analysis.

At a patient level, written responses regarding ‘Do you experience jaw pain, grinding and/or clenching and if so, how does this affect your quality of life?' were also recorded as qualitative data which was transformed into a quantitative code through data categorisation (see Table 4) and then subject to analysis via chi-square tests.

### 2.4. Ethical Considerations

Ethical approval was granted by the Griffith University Human Research Ethics Committee (GU Ref No: 2023/780) on the 26th of September 2023 prior to the commencement of any data collection. Ethical considerations regarding student, patient and dental practitioner privacy were all assessed and deemed to meet satisfactory standards. Each questionnaire was carefully designed in accordance with ethical guidelines and was deemed appropriate before trial testing the questionnaire.

Questionnaires were distributed in the GUDC, targeting students during their clinical sessions, patients in the waiting areas before and after treatment, and dental practitioners between clinical supervision sessions. Before the survey, the study's nature, risks and benefits were disclosed to participants, ensuring voluntary participation and informed consent. Responses were then collected, de-identified, and securely stored.

## 3. Results

### 3.1. Demographic Data

The questionnaire was completed by a total of 325 participants (171 patients, 130 students and 24 dental practitioners) within the GUDC, Australia. The details of the participants' demographic data are summarised in Table 2.

Group-specific demographic data was also collected. Patient education level was recorded, and students were asked to state their year level. Dental practitioners were asked about their years of experience and specialty. Experience in years (range 5–47) was grouped below 10, 10–19, 20–29 and above 30. Finally, dental practitioners were also asked about their specialty. The group-specific demographic data is summarised in Table 5.

### 3.2. Patients' Questionnaire

Chi-square tests of patient data indicated that *age* had no significant effect on participants' awareness of non-cosmetic BTX treatments (*p*=0.10), previously received BTX (*p*=0.31), willingness to receive BTX (*p*=0.37), side effects (*p*=0.06), duration of effect (*p*=0.64), awareness of TMD (*p*=0.98) or experience of TMD (*p*=0.09). Similarly, education level had no significant effect on awareness of non-cosmetic BTX treatments (*p*=0.96), previously received BTX (*p*=0.88), willingness to receive BTX (*p*=0.90), side effects (*p*=0.39) and duration of effect (*p*=0.21).

**Figure 1:**
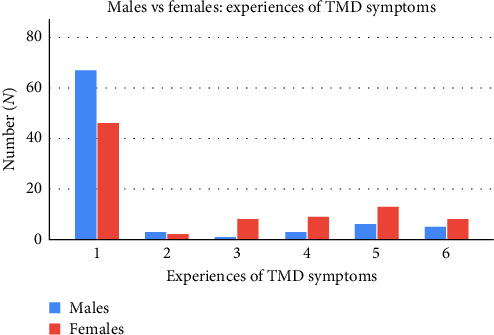
Experience of TMD symptoms for males vs. females. Histogram of patients' experience of TMD. Responses are from males (blue) vs. females (red). Codes for patient responses were as follows; 1 = Nil symptoms or issues reported, 2 = quality of life not affected: symptoms reported but no impact on quality of life, 3 = Jaw pain as the primary symptom, 4 = clenching/grinding only: Reports of clenching or grinding without jaw pain, 5 = combined symptoms: Combination of jaw pain with clenching, grinding or other milder symptoms including tooth wear, 6 = significant impact: symptoms that significantly impact quality of life (e.g., headaches, tinnitus, sleep apnoea, can't open jaw fully, tooth loss).

However, the analysis determined that gender had a significant effect on the awareness of non-cosmetic BTX treatments (*p*=0.02), with a slightly higher proportion of females (38.40%) being more aware than males (36.50%). Furthermore, there was a statistically significant association between gender and previously received BTX (*p*  < 0.001), with a small number of females (15.10%) answering yes, while all males (0.00%) answered no. Similarly, gender had a significant effect on willingness to receive BTX (*p*=0.02), as females (54.70%) were more willing to receive BTX than males (36.50%). Gender also had a significant effect on awareness of BTX side effects (*p*=0.02) and the duration of effect (*p*  < 0.001). Females showed slightly higher awareness of *BTX* side effects (20.90%) and the duration of effect (27.90%) compared to males, who had awareness levels of 8.20% and 5.90%, respectively. Furthermore, gender and education level both had a significant effect on participants' awareness of TMD symptoms (*p*=0.01 and 0.01, respectively). A small percentage of males (8.20%) were aware of TMD symptoms when compared to females (22.10%). In regard to education level, those who completed a bachelor degree reported the most awareness of TMD symptoms (34.50%), followed by high school (12.60%), vocational training (8.30%) and master's courses (0.00%). Similarly, gender also had a significant effect on patients' experience of TMD (*p*=0.007), with most males reporting no symptoms (78.80%) and those who have reported combined symptoms (7.10%) and significant impacts on life (5.90%). Conversely, females appeared to experience more TMD, with approximately half reporting no symptoms (53.50%), followed by combined symptoms (15.10%), clenching and/or grinding (10.50%), jaw pain as the primary symptom (9.30%) and significant impacts on life (9.30%; Figure 1).

### 3.3. Students' Questionnaire

Chi-square tests were conducted to evaluate the influence of gender, age and year level on various aspects of students' awareness and knowledge relating to BTX. The results demonstrate that gender, age and year level had no significant effect on awareness of BTX side effects (*p*=0.11, 0.16, 0.81, respectively), their intention to administer BTX post-graduation (*p*=0.15, 0.56, 0.15, respectively), or the incorporation of BTX into undergraduate studies (*p*=1.00, 0.91, 0.13, respectively). Moreover, gender (*p*=0.44) and age (*p*=0.71) did not significantly affect students' knowledge of non-cosmetic BTX treatments. Similarly, no significant relationship was found between gender (*p*=0.54) and year level (*p*=0.13), and awareness of the duration of BTX effects. Additionally, age (*p*=0.52) and year level (*p*=0.32) did not significantly influence knowledge of BTX synthesis, and gender (*p*=0.76) had no significant effect on knowledge of BTX dosage. Last, there was no significant association between gender (*p*=0.34) or age (*p*=0.16) and students' preferred treatment options.

**Figure 2:**
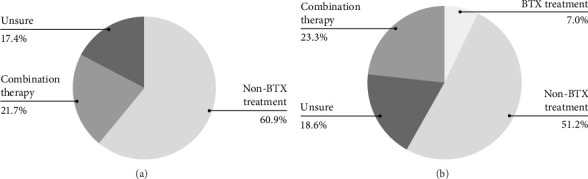
Preferred treatment options for dental practitioners and students. Note. Pie chart (a) illustrates GU dental practitioners' preferred treatment options for TMD and bruxism. Data revealed that 60.90% preferred non-BTX treatment, 21.70% preferred combination therapy with 17.40% were unsure of treatment options for TMD. Pie chart (b) illustrates the responses of GU students' preferred treatment options for TMD and bruxism. Data revealed that 51.20% preferred non-BTX treatment, 23.30% preferred combination therapy, 7.00% would prefer BTX treatment and 18.60% were unsure of treatment options for TMD.

Several statistically significant effects were observed across variables related to students' knowledge and awareness of BTX. Age (*p*=0.002) and year level (*p*=0.001) were significantly associated with knowledge of BTX dosage. The highest awareness was associated with 40–49 (100%) year-olds, followed by 20–29 (13.8%) and 30–39 (8.3%) year-olds. Awareness also significantly increased with year level as fifth-year (27.3%) students ranked highest and then fourth-year (8.3%) and third-years (0%). Furthermore, year level (*p*=0.002) significantly influenced awareness of non-cosmetic BTX treatments, again with knowledge of treatment increasing from third to fifth (77.8%, 83.3%, 100%, respectively) year of study. While *age* (*p*=0.02) significantly impacted knowledge of the duration of BTX effects, with older students between the ages of 40–49 (100%) having more knowledge than 30–39-year-olds (66.7%) and 20–29-year-olds (32.5%). Furthermore, gender (*p*=0.003) significantly influenced the knowledge of BTX synthesis, with males (40.8%) exhibiting more awareness compared to females (16.9%). Additionally, year level significantly affected students' preferred treatment (*p*  < 0.001). Most third-year students were unsure (63%), while most fourth- and fifth-year students preferred non-BTX treatments (64.6% and 58.2%, respectively), with a smaller proportion of fifth-year students favouring combination treatments (30.9%). The overall preferred treatment options have been illustrated as shown in Figure 2b.

### 3.4. Dental Practitioners' Questionnaire

The chi-squared test was used to analyse whether dental practitioners' demographic data such as gender, age, experience, or specialty had any effect on their awareness of BTX for treatment of TMD. The analysis revealed that these variables had no significant effect on knowledge of non-cosmetic BTX treatments (*p*=0.85, 0.78, 0.37, 0.91, respectively), receiving BTX training (*p*=0.57, 0.36, 0.49, 0.49, respectively), having administered BTX (*p*=0.27, 0.45, 0.77, 0.63, respectively), knowledge on duration of effect (*p*=0.36, 0.84, 0.43, 0.20, respectively), knowledge of dosage (*p*=0.09, 0.83, 0.45, 0.93, respectively), knowledge of synthesis (*p*=1.00, 0.69, 0.68, 0.39, respectively), confidence in administering (*p*=0.44, 0.07, 0.66, 0.38, respectively), offered in workplace (*p*=0.96, 0.86, 0.88, 0.12, respectively) and preferred treatment for TMD (*p*=0.85, 0.88, 0.50, 0.86, respectively). The overall preferred treatment options are illustrated in Figure 2, section 2a. It was also found that age (*p*=0.70), gender (*p*=0.46), and experience (*p*=0.54) had no significant effect on dental practitioners' awareness of BTX side effects. However, there was a statistically significant finding relating to dental practitioners' specialty and their awareness in regard to the side effects of BTX (*p*=0.01). Within specialties, all prosthodontics (100.00%) and periodontists (100.00%) were aware of the side effects of BTX, while no oral surgeons (0.00%) were aware of the side effects. Following this, almost all non-specialised dental practitioners were aware of BTX side effects (93.80%).

### 3.5. Comparative Analysis of Questionnaires From Patients, Students and Dental Practitioners

The chi-squared analysis examined the differences between the groups of patients, students, and dental practitioners across similar survey questions. Statistical analysis suggested a significant difference between groups regarding their knowledge of non-cosmetic uses (*p*  < 0.001). Most dental practitioners (95.80%) and students (89.20%) had more awareness regarding non-cosmetic treatment when compared to patients (30.40%). Furthermore, there was a statistical difference between groups regarding their knowledge of side effects (*p*  < 0.001), with a large proportion of dentists (91.70%) being aware of BTX side effects, followed by half of the students (50.00%) and a small percentage of patients (14.60%). Additionally, there was a significant difference when it comes to awareness of the duration of effect (*p*  < 0.001) depending on the group, with dental practitioners being the most informed (79.20%), compared to a smaller proportion of students (38.50%) and patients (17.00%).

## 4. Discussion

This study aimed to collect data via surveys at GUDC to assess the awareness and attitude towards the therapeutic use of BTX for the treatment of TMD and bruxism. In total, 325 individuals participated, consisting of dental students, dental patients and dental practitioners at GU. The results provide insight into the current knowledge and attitudes of these groups, and the findings from this study have important implications for both theoretical understanding and practical application in dental education and clinical practice. Assessment of awareness and knowledge of dental students, dental practitioners and dental patients provides invaluable information to determine future courses of action required to improve the general Australian understanding and perception of BTX and its possible uses. The findings of this research prompted the need for further educational programmes within the dental community. The development of knowledge surrounding this topic will lead to enhanced patient education, treatment options, interdisciplinary collaboration, policy development, research opportunities and market growth. Furthermore, by including and analysing demographic variables in the statistical analysis, this could ultimately provide for a more targeted approach to improving BTX awareness levels.

Studies on BTX awareness using questionnaires found that dental students have a general knowledge of BTX use in aesthetics [22–26]. Despite this established general awareness, 89% of dental students across the UK reported they had no prior teachings on BTX treatment, and additionally, 38% of students in Chennai reported being unaware of the risks associated with BTX treatment [23, 25]. Furthermore, a cross-sectional study investigated the knowledge and attitude of BTX use among Saudi and Pakistani dentists. The data illustrated that both Saudi and Pakistani dentists had adequate knowledge of BTX use for aesthetics (54.7%) and its side effects (66.8%). However, it was concluded that amongst dentists from both countries, only 3.51% of dentists practised using BTX, suggesting a gap in knowledge or unwillingness to adopt BTX as a therapeutic treatment [27]. A similar study in Thailand [28], found a smaller proportion of dentists had adequate knowledge of BTX use in dentistry (27.5%) when compared to the study by Sulaiman et al. [27] (54.7%). However, Thai dentists showed more willingness to use BTX clinically, with 16% of dentists previously treating patients with BTX [28]. By contrast, another similar study conducted on 245 Saudi dentists reported only 51.8% and 44.5% were aware of BTX indications for TMD pain and bruxism, respectively [29]. The lack of awareness surrounding BTX and its therapeutic use within dentistry was also shown to be lacking in another study of 150 dentists found that only 31.6% were aware of its use for TMD, and only 21.5% were aware of its uses in jaw-clenching patients [30]. These studies, however, may prove to be an inaccurate representation of both awareness and practice of BTX within Australian communities, suggesting further research for an Australian demographic is required.

The significance of this study lies in its potential to bridge the knowledge gap regarding BTX's therapeutic application in managing TMD and bruxism. The first step in this process is to assess the awareness levels regarding various aspects of BTX among Australian dental practitioners, dental students and patients. The study highlights the knowledge gap surrounding certain applications of BTX within the various groups. Although the data indicates that BTX is well-known for cosmetic purposes, its use to manage bruxism and TMD is less understood, particularly among patients, and to a lesser extent amongst students. This finding aligns with current literature that has emphasised that dental students and dental practitioners have limited knowledge regarding BTX's therapeutic value beyond cosmetic purposes, despite growing evidence supporting the use of BTX in managing orofacial pain and dysfunction [20]. Also, Freeman et al. [22] demonstrated the lack of knowledge surrounding BTX use beyond cosmetic purposes, as almost all patient participants answered that the only application of BTX was for aesthetic enhancement. Studies on BTX awareness using questionnaires found that dental students generally understand its aesthetic applications [23–26]. However, most have not received formal education on therapeutic uses, with almost 90% of dental students across the UK reporting no prior teachings on BTX treatment [23].

Students, particularly those in earlier years, showed limited knowledge of BTX for non-cosmetic purposes, with third-year students being the least aware compared to fourth- and fifth-year students. Furthermore, statistically significant findings were seen for knowledge of dosage and preferred treatment for TMD between year level for the student population group. Although most students lacked knowledge of the therapeutic dosage of BTX, as students progressed from third to fifth-year their knowledge improved. Similarly, students in their fifth-year were more likely to offer combination treatment for TMD, compared to their third-year counterparts. This association between knowledge and year level is also observed in a Malaysian university study, demonstrating increased self-reported knowledge of prevention and detection of oral cancer as year level increases amongst dental students [31]. This finding may imply that as the students progress through their dental degree, their knowledge of treatment options may improve [31]. It should be noted that the topic of TMDs is first taught to dental students at Griffith University in the third year of the programme by academics who are general dental practitioners, oral surgeons and prosthodontists, while BTX applications is not taught at all as part of the undergraduate curriculum. We believe that the curriculum design within the educational facility where the study was conducted would influence our results. Furthermore, almost half of the student population preferred non-BTX treatment for TMD (see Figure 2, section 2b). This preference may be due to a lack of BTX knowledge and the university clinic not offering BTX treatment for patients. Further, interesting findings were observed when analysing the demographic variables, age and gender in the student population. Despite the overall lack of awareness from both genders in regard to the synthesis of BTX, males demonstrated more awareness. Alfouzan and Mekkawy [29] reported similar findings in regard to the overall lack of synthesis knowledge; however, no significant difference was displayed between the two genders. This may suggest further research is necessary to fully elucidate the extent and nature of gender's role in shaping knowledge about BTX.

With age, 20–29-year-olds had greater knowledge of synthesis and duration of effect compared to 30–49-year-olds. As a large proportion of the student population are between 20–29-year-old, this may be a possible explanation for the findings. Freeman et al. [22] report that 19–30-year-olds receive the most BTX injections and receive their first injection earlier than older age groups did. This study also highlighted a notable increase in the younger demographic populations receiving BTX compared to previous generations of people who have received the drug [22]. This may provide an explanation for younger participants having greater BTX knowledge. Moreover, an extensive proportion of this population would like to receive further education on BTX and plan to offer BTX treatment following graduation. This is supported by Al Hamdan et al. [30], as many participating dental practitioners would like to be able to deliver BTX for various reasons. In turn, this could inadvertently increase the awareness within the patient population, as improved dental practitioner knowledge of dental treatment options is shown to translate to improved patient awareness, knowledge and treatment outcomes. This transmission of information was observed in a study about implants, which included a similar population of low-socioeconomic patients [32]. Overall, these findings reinforce the need for the educational curriculum to include the use of BTX for therapeutic purposes.

Through analysis of patient data, statistically significant differences in survey responses between genders were identified. Female patients received BTX more, were more aware of the duration of effect, non-cosmetic uses and side effects, and reported higher willingness to receive BTX than their male counterparts. This was strongly reflected by Alfouzan and Mekkawy [29], who established females had a higher awareness of non-cosmetic uses and side effects. The results found that more females have received BTX previously, possibly improving their general knowledge of the drug's profile and consequently positively influencing future willingness to receive BTX. Literature suggests that more females receive BTX than their male counterparts, and the most common reason is for improvement of self-esteem and appearance [33]. In addition, female patients reported increased awareness of TMD symptoms and had more experience of TMD than males. This is supported by Schmidt et al. [34], who reported that more females than male participants reported at least one TMD symptom in a self-reported questionnaire. Furthermore, Barsky et al. [35] state that females have increased awareness of disease symptoms, report more intense symptoms and require more profound symptomatic treatment for conditions, almost mirroring the results observed in this study. Since the questionnaire did not specify the reason for BTX administration, those participants may have received BTX for treatment of TMD and bruxism, resulting in selection bias of the sample and a reported increased awareness of TMD symptoms and TMD experience. Further research should specify why BTX was administered to further investigate this correlation.

In a practical sense, this study identifies further areas where dental education and clinical training can be directed. Although patients and students lacked knowledge regarding the use of BTX for TMD and bruxism, a higher proportion of dental practitioners demonstrated a greater understanding of its therapeutic use. Recent studies have found that dentists have a higher level of knowledge about oral cancers compared to senior dental students [36]. Furthermore, another study also conducted at GUDC found that dental students had greater awareness of certain risk factors for oral cancer when compared to patients [37]. These findings align with the trends observed within this research paper. Overall, this may indicate a potential gap in the effective transfer of information from practitioners and students to patients, indicating that more effort may be needed to ensure patients are adequately informed.

Within the specialist group, prosthodontists and periodontists had greater awareness relating to BTX side effects, compared to oral surgeons. While the current literature lacks data regarding dental specialty and their respective knowledge regarding BTX, a questionnaire assessing COVID-19 knowledge reported no differences among different specialties [38]. Therefore, these findings may not be sufficient to draw accurate conclusions due to the small sample size within this group, specifically as only seven specialists participated, with only one oral surgeon partaking. This needs to be further investigated, particularly as an international study found that 51% of physicians were unfamiliar with potential contradictions, and 26% were unaware of non-cosmetic indications for BTX [39]. To direct future education, subsequent studies should aim to gather a greater specialist population to improve internal and external validity [40]. Furthermore, a high percentage of the dental practitioner population have received no training in delivering BTX and did not offer BTX treatment within their workplace, translating to reduced confidence in administering BTX. This is corroborated by Al Hamdan et al. [30], as most dental practitioners reported a lack of knowledge and experience as the main reason they lacked confidence in delivering BTX. The oral surgeon's lack of training and exposure to BTX treatment may explain the lack of comprehension. This is contradictory to current literature, as BTX is commonly used by oral surgeons to assist recovery and pain management following oral surgeries [41]. Due to the lack of knowledge surrounding the practical uses of BTX seen in literature [30], a large proportion of dental practitioners preferred to use a non-BTX treatment modality regarding TMD or were unsure about appropriate treatment modalities for TMDs (see Figure 2, section 2a). This could be attributed to the fact that were more familiar with other treatment modalities in comparison to BTX. The results from the present study support the need for a higher focus in dental schools regarding the teaching of orofacial pain which falls under the umbrella of oral medicine rather than being a standalone speciality in Australia. It also highlights the importance of recruiting oral medicine and/or orofacial pain specialists as academics in universities. It should be noted that the choice of our quantitative codes was in alignment with previous literature that classified therapies for TMDs as either involving BTX versus 'other' therapies which is consistent with our terminology of BTX versus non-BTX [42].

Despite the widespread use of BTX in cosmetic procedures and the wide acceptance of BTX in aesthetic applications in Australia, the application of BTX for the management of TMDs is not covered through the public system (Medicare) with the exception of a small percentage of cases where the patients are under the care of a specialist oral and maxillofacial surgeon. The Dental Board of Australia has a fact sheet about the use of botulinum toxin and dermal fillers by dentists which reports that a specific policy is not required for this type of procedure because the existing regulatory framework, which includes the Board's standards, codes and guidelines, applies to all dental practitioners regardless of the type of care being delivered. Furthermore, all dental practitioners in Australia are expected to practice within the scope of their education, training and competence. As the application of BTX is not taught in undergraduate curricula, a number of continuing professional development courses are available for dental practitioners, some of which are coordinated and delivered by the Australian Dental Association (Australia). We believe that the regulatory and legal standpoint as well as the public perception in Australia would have an impact on the results of the current in comparison to other studies that were conducted in a different part of the world with a different regulatory framework.

The demographic data results from the current study were representative of the patient, students and dental practitioners' population that attend the GUDC. Students were excluded from participating in the study if they were a first or a second-year dental student due to not commencing clinical practice under supervision in the university's dental clinic nor receiving any didactic education about the topic of TMDs yet. Furthermore, the patient population seen at the GUDC, being predominantly sourced from the public system, is swayed towards older patient/pensioners with lower socioeconomic and educational status. However, some private patients still attend the university clinic, which was reflected in the demographic data. This is corroborated by Regidor et al. [43] who determined that patients attending private health clinics, such as non-government-funded dental practices, had a higher socioeconomic status. Tsang [40] stated that differences in demographic factors between populations can reduce the external validity of research findings. However, this study likely represents other universities more accurately than the general dental public, due to similarities across dental curriculums and similar socioeconomic status of government-funded patients. Regidor et al. [43] demonstrated that the demographic attending public health settings, such as university clinics, consisted primarily of low-socioeconomic patients. Additionally, the utilisation of the chi-square test allowed robust statistical analysis within and between dental practitioners, students and patients while also considering demographic data. Chi-square tests allowed identical questions across group-specific questionnaires to be analysed for correlations [44], providing research that was currently lacking in the literature.

A limitation of the study is the small sample size of dental practitioners. With only about 40 general dental practitioners and 15 specialists within the GUDC. It was not possible to include all dental practitioners due to differing schedules, with six dental practitioners also declining participation. Furthermore, GUDC employs a relatively small number of dental specialists and lacks certain specialties. The small sample size of dental specialists may provide an inaccurate representation of this population, and the data may not represent all specialties, however, it could be considered a valid representation of the GUDC population. Moreover, various questionnaire responses rely on self-reported awareness for completion. This may introduce bias, as participants may over- or under-estimate their awareness levels, decreasing the validity and accuracy of data if they do so [45]. Furthermore, standardisation of the questionnaires would help replication of the study at an international level and extend the possibilities to achieve findings that are applicable at a global level.

Future longitudinal studies could be designed to assess the improvement of BTX knowledge and awareness from baseline after implementation of specific BTX educational programmes for dental practitioners, students and patients. The results may also determine the correlation between improved education and treatment acceptance levels relating to BTX. A future pathway that this study may provide is the integration of BTX-specific teachings for students and practitioners, with the addition of potential community-based education such as pamphlets in clinic waiting rooms which may appeal to patients suffering from TMD and bruxism.

## 5. Conclusion

The current study established that there is a significant difference in the awareness of BTX between the groups surveyed, with patients displaying the least awareness and dental practitioners being most aware. Furthermore, demographic data also impacted these differences, with the patient's gender influencing their awareness and the student's age, gender and year level all impacting their responses significantly. Future research involving a large cohort of specialists was deemed necessary to facilitate more accurate comparisons. Targeted educational teachings within university curricula and patient education campaigns could address the identified deficiencies in BTX awareness. Such initiatives could lead to improved patient education regarding treatment options, guidelines and ultimately enhanced patient outcomes.

## Figures and Tables

**Table 1:** Summary of questionnaires given to patients, students and dental practitioners with respective question types in brackets.

Patient	Student	Dental practitioner
Age? (written response)	Age? (written response)	Age? (written response)
Gender? (written response)	Gender? (written response)	Gender? (written response)
Highest level of education? (written response)	Year level? (written response)	Years of experience as a dental practitioner? (written response)
Are you aware of the use of botulinum toxin for other treatments besides cosmetic purposes? (yes/no)	Are you aware of the use of botulinum toxin for other treatments besides cosmetic purposes? (yes/no)	If applicable, what field do you specialise in? (written response)
Have you ever received botulinum toxin injections? (yes/no)	Would you like to incorporate botulinum toxin into your undergraduate clinical studies? (yes/no)	Are you aware of the use of botulinum toxin for other treatments besides cosmetic purposes? (yes/no)
I am willing to receive botulinum toxin treatment for TMD and Bruxism. (yes/no)	Would you consider providing botulinum toxin treatment after graduation? (yes/no)	Have you ever received any training to deliver botulinum toxin injections? (yes/no)
Are you aware of the side effects of botulinum toxin treatment? (yes/no)	Are you aware of the side effects of botulinum toxin treatment? (yes/no)	Have you ever given therapeutic botulinum toxin injections to your patients? (yes/no)
Are you aware of how long botulinum toxin may last? (yes/no)	Are you aware of how long botulinum toxin may last? (yes/no)	Are you aware of the side effects of botulinum toxin treatment? (yes/no)
Are you aware of the symptoms regarding Temporomandibular disorders (TMD) and/or Bruxism? (yes/no)	What is the recommended dosage for therapeutic botulinum toxin use in TMD? (written response)	Are you aware of how long botulinum toxin may last for? (yes/no)
Do you experience jaw pain, grinding and/or clenching and if so, how does this affect your quality of life? (Written response)	Are you aware of how botulinum toxin is derived? (yes/no)	Are you aware of the recommended dosage for botulinum toxin? (yes/no)
	What treatment would you prefer for TMD and bruxism and why? (written response)	Are you aware of how botulinum toxin is derived? (yes/no)
	—	Would you feel comfortable delivering botulinum toxin? (strongly disagree, disagree, neutral, agree, strongly agree)
	—	Does your workplace offer botulinum toxin treatment for Temporomandibular disorders (TMDs) or bruxism? (yes/no)
	—	What treatment would you prefer for TMD and bruxism and why? (written response)

*Note*: The questionnaires also included a demographic section. The questions were either dichotomous, written responses, or a Likert-Scale. The demographic section obtained participants' age, and gender across all questionnaires, with years of experience, education level, and year level as applicable.

**Table 2:** Demographic data of patients, students and dental practitioners.

Demographic data	Patients (*N* = 171), *n* (%)	Students (*N* = 130), *n* (%)	Dental practitioners (*N* = 24), *n* (%)	Total (*N* = 325), *n* (%)
Age				
<20	1 (0.59)	0 (0.00)	0 (0.00)	1 (0.31)
20–29	6 (3.51)	116 (89.23)	0 (0.00)	122 (37.54)
30–39	12 (7.02)	12 (9.23)	7 (29.17)	31 (9.54)
40–49	11 (6.43)	2 (1.54)	9 (37.50)	22 (6.77)
50–59	27 (15.79)	0 (0.00)	2 (8.33)	29 (8.92)
60>	114 (66.67)	0 (0.00)	6 (25.00)	120 (36.92)
Gender				
Male	85 (49.71)	71 (54.61)	15 (62.5)	171 (52.62)
Female	86 (50.29)	59 (45.39)	9 (37.5)	154 (47.38)

*Note*: The table presents age and gender in numerical and percentage formats.

**Table 3:** Examples of students' and dental practitioners' qualitative data and their respective quantitative codes.

Open-ended question response	Quantitative code
‘Botulinum toxin'	1 - BTX
**‘**Splint,' ‘mouthguard'	2 - Non-BTX treatment
**‘**Splint, physical therapy and botulinum toxin' ‘splint and botulinum toxin'	3 - Combination treatment
**‘**I don't know'	4 - Unsure

**Table 4:** Examples of patients' qualitative data and their respective quantitative codes.

Open-ended question response	Quantitative code
‘No pain,' ‘nil'	1 - Nil symptoms or issues reported
‘Bit of pain sometimes, but it does not affect my quality of life'	2 - Quality of life not affected: symptoms reported but no impact on quality of life
‘Jaw pain'	3 - Jaw pain is the primary symptom
‘I grind my teeth,' ‘I clench when I am stressed'	4 - Clenching/Grinding only: reports of clenching or grinding without jaw pain.
‘Grinding and tooth wear'	5 - Combined symptoms: combination of jaw pain with clenching, grinding, or other milder symptoms including tooth wear
‘Can't open fully,' ‘loss of sleep,' ‘tinnitus and tooth loss'	6 - Significant impact: symptoms that significantly impact the quality of life (e.g., headaches, tinnitus, sleep apnoea, can't open the jaw fully, tooth loss)

**Table 5:** Demographic data obtained from patients, students, and dental practitioners.

Patients	*N* (%)
Education level	
High school	111 (64.91%)
Bachelor	29 (16.96%)
Vocational	24 (14.04%)
Masters	7 (4.09%)
Students	
Year level	
3 (3rd year of bachelor of dental health science)	27 (20.77%)
4 (1st year masters of dentistry)	48 (36.92%)
5 (2nd year masters of dentistry)	55 (42.31%)
Dental practitioners	
Experience (years)	
<10	6 (25.00%)
10–19	6 (25.00%)
20–29	6 (25.00%)
30>	6 (25.00%)
Speciality	
None	16 (66.67%)
Yes	8 (33.33%)
Prosthodontics	4 (16.67%)
Periodontics	3 (12.5%)
Oral surgery	1 (4.17%)

*Note*: Educational level was obtained from patients and has been categorised as either high school, bachelor, vocational or masters. Students were required to state the year level they are in as either third-, fourth- or fifth-year dental students. Dental practitioners were asked about their years of experience and specialty. The data collected has been presented in table format with the percentages and participant numbers having been included.

## Data Availability

The data that support the findings of this study are available from the corresponding author upon reasonable request.
